# Miscellaneous Medical Intelligence

**Published:** 1849

**Authors:** 


### ARTICLE VI.

### MISCELLANEOUS MEDICAL INTELLIGENCE.

Dr. J. Blount of Lockport asks: — If it is generally known that unguentum hydrargyri, can be made with a mere tithe of the labor that is usually given to it, and says, "I prepare it by substituting spermaciti for axunge.

"Rub the hyd. with the spermaciti a few minutes, add a few drops of sweet oil, stir a *minute* and you have a good hard neat wax, in the place of the soft offensive article formerly used."

We hear that a State Medical Convention is to meet at Indianapolis, by the call of the Indianapolis Medical Society about the first of June next, but cannot learn the day appointed.

Dr. Drake, than whom few men enjoy a higher reputation for extensive professional learning, and for ability as a sound and practical teacher, has retired from his chair in the Univeristy of Louisville, and has announced himself for practice in Cincinnati.

We hope that the leisure from the duties of his chair will enable him to complete the long promised and anxiously looked for work upon the practice of medicine in the West. A work much called for by the wants of the profession, and which the long experience and devotion to the subject of Prof. Drake, eminently qualify him to produce.

We regret to learn of the death of J. Harrison, M.D., late Editor of the ably conducted periodical, the New Orleans Med. and Surg. Jour., and Professor of Physiology and Pathology in the University of Louisiana. In him the medical profession has lost a talented writer, and an able and popular teacher.

